# *Gastrochilus immaculatus* (Orchidaceae, Epidendroideae), a new epiphytic orchid from Chongqing, Southwest China

**DOI:** 10.3897/phytokeys.280.206936

**Published:** 2026-10-19

**Authors:** Jun-Yi Zhang, Min Liao, Pan-Yan Yang, Yue-Hong Cheng, Jin-Zhong Wu, Peng-Cheng Zhang, Ding-Xiang Yu, Feng Chen, Bo Xu

**Affiliations:** 1 China-Croatia Belt and Road Joint Laboratory on Biodiversity and Ecosystem Services, Mountain Ecological Restoration and Biodiversity Conservation Key Laboratory of Sichuan Province, Chengdu Institute of Biology, Chinese Academy of Sciences, Chengdu 610213, China China-Croatia Belt and Road Joint Laboratory on Biodiversity and Ecosystem Services, Mountain Ecological Restoration and Biodiversity Conservation Key Laboratory of Sichuan Province, Chengdu Institute of Biology, Chinese Academy of Sciences Chengdu China; 2 College of Life Sciences, Sichuan University, Chengdu 610064, China College of Life Sciences, Sichuan University Chengdu China; 3 University of Chinese Academy of Sciences, Beijing 10049, China University of Chinese Academy of Sciences Beijing China; 4 Wolong National Natural Reserve Administration Bureau, Wenchuan 623006, China Wolong National Natural Reserve Administration Bureau Wenchuan China; 5 Chongqing Agricultural Ecology and Resource Protection Station, Chongqing 401121, China Chongqing Agricultural Ecology and Resource Protection Station Chongqing China; 6 Chongqing Academy of Agriculture Sciences, Chongqing 401329, China Chongqing Academy of Agriculture Sciences Chongqing China; 7 Chongqing Museum of Natural History, Chongqing 400799, China Chongqing Museum of Natural History Chongqing China

**Keywords:** Chongqing, *
Gastrochilus
*, new species, Orchidaceae, phylogeny

## Abstract

*Gastrochilus immaculatus* (Orchidaceae) is described and illustrated as a new species from Chongqing, southwestern China, where it grows epiphytically on tree trunks or rock walls in evergreen broad-leaved forests. It differs from the morphologically similar *G. fargesii* by its shorter stems, broader falcate-oblong leaves, and, especially, its golden-yellow immaculate flowers with a rather acute apex of the hypochile. Phylogenetic analyses based on nuclear ITS and four chloroplast markers (*mat*K, *psb*A–*trn*H, *psb*M–*trn*D, and *trn*L–F) strongly support it as a sister species of *G. fargesii* within *G.* sect. *Acinacifolii*. Only three small subpopulations were discovered within a scenic spot in a deep limestone valley in southern Chongqing, warranting a preliminary IUCN classification of Critically Endangered (CR). This discovery also highlights the overlooked epiphytic orchid diversity along the mountain ranges surrounding the Sichuan Basin.

## Introduction

The epiphytic orchid genus *Gastrochilus* D.Don (Don 1825) comprises approximately 89 species distributed from tropical to warm-temperate Asia (Tsi 1996, 1999; Liu et al. 2019; Govaerts et al. 2021; Zhou et al. 2021; Zhang et al. 2022; Ya et al. 2023; Zhang et al. 2024a, 2024b; Xiong et al. 2025; POWO 2026). Members of the genus are readily recognized by their saccate hypochile that forms a distinct spur, together with two sub-globose pollinia attached to a slender stipe (Pridgeon et al. 2014; Zhang et al. 2024a). Most species were discovered in the past 20 years, and more than 20 species were described in the most recent 5 years, particularly from the regions adjacent to the Sichuan Basin (Liu et al. 2019; Rao et al. 2019; Wu et al. 2019; Liao et al. 2022; Nguyen et al. 2022; Zhang et al. 2022; Lee et al. 2023; Liu et al. 2023; Ya et al. 2023; Zhang et al. 2024a; Zhang et al. 2024b; Zhou et al. 2024; Xiong et al. 2025; Zhu et al. 2025). With the accumulated molecular data and morphological evidence, this genus was subdivided into six sections (Zhang et al. 2024a).

**Figure 1.**
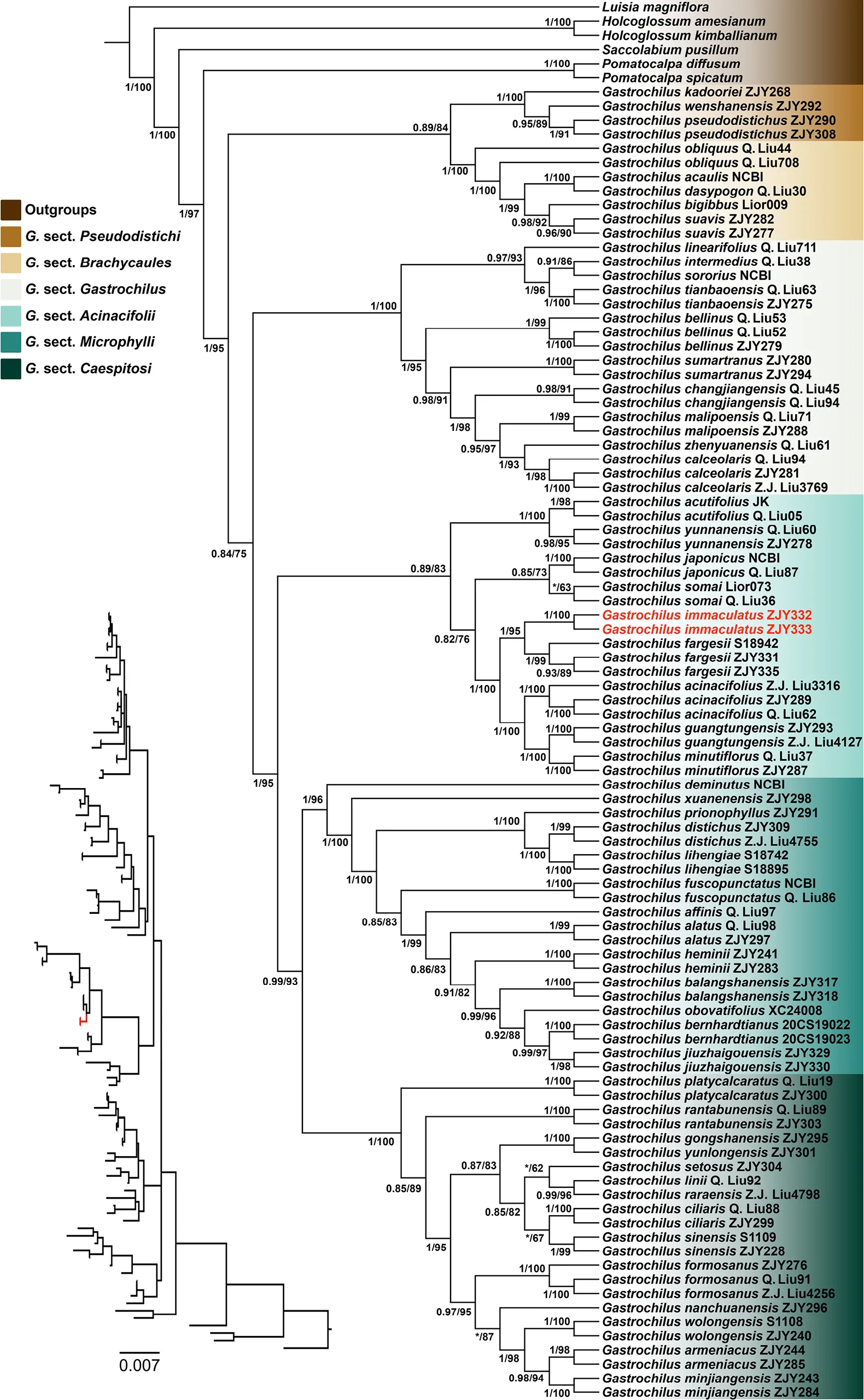
Maximum likelihood phylogenetic tree of *Gastrochilus*, including 60 taxa, based on the combined nrITS and four plastid markers (*mat*K, *psb*A–*trn*H, *psb*M–*trn*D, and *trn*L–F). Values before the slash indicate Bayesian posterior probabilities, and numbers after the slash indicate ML bootstrap support values for major lineages. An asterisk (*) indicates that a node is not supported in the analysis. The two accessions of the inferred new species are highlighted in red, and the colors of the terminal nodes correspond to the six sections of *Gastrochilus* defined in Zhang et al. (2024a).

In September 2025, during a survey in Qijiang District, Chongqing, Southwest China, an unusual flowering *Gastrochilus* species was encountered in an evergreen broad-leaved forest at elevations between 800 and 1,100 m. The locality is close to Jinfo Mountain in Nanchuan District, Chongqing (Fig. 1), where Yi et al. (2010) collected and described *G. brevifimbriatus* S.R. Yi (=*G. fargesii* (Kraenzl.) Schltr. according to Liu et al. 2023). This orchid also resembles *G. fargesii*, which is a member of *G.* sect. *Acinacifolii* Q. Liu & J.Y. Gao ex Jun Y. Zhang & H. He, characterized by falcate-oblong leaves and a subtriangular epichile with a central thickened cushion, an adaxially glabrous surface, and a lacerate or erose in margin (Zhang et al. 2024a). However, it showed a remarkable difference in flower color, being yellow without deeper spots. Based on previous studies (Liao et al. 2022; Zhang et al. 2022; Zhang et al. 2024a, 2024b; Zhu et al. 2025), this species is here named *G. immaculatus*, with the hypothesis that it is a new member of the same section as *G. fargesii*.

## Materials and methods

### Morphological analyses

Field collections of herbarium specimens and silica-gel-dried leaves of *Gastrochilus immaculatus* were carried out in Heishan Valley, Qijiang District, Chongqing, Southwest China. Morphological observations and measurements were performed on both living plants and dried voucher specimens, which are deposited at CDBI (herbarium acronyms follow Thiers 2026). Descriptive terminology followed Beentje (2016), and the description order and arrangement referred to Chen et al. (2009).

### DNA extraction, amplification, and sequencing

For *Gastrochilus immaculatus*, leaf samples from two individuals (collected from two different subpopulations) were used, and for *G. fargesii*, three accessions were included: *S18942* (from Ya et al. 2023) and two additional samples (*ZJY331* and *ZJY335*) added in this study. Total DNA was extracted exclusively from silica-gel-dried leaves using a Plant DNA Isolation Kit (Cat. No. DE-06111). Following the phylogenetic protocols for *Gastrochilus* established by Liu et al. (2019) and Zhang et al. (2024a), the same primer sets were employed to amplify the nuclear ribosomal internal transcribed spacer (nrITS) and four chloroplast markers (*mat*K, *psb*A–*trn*H, *psb*M–*trn*D, and *trn*L–F) via polymerase chain reaction (PCR). All amplified products were sequenced by TSINGKE Biotech Co. Ltd. (Chengdu, China). The manually edited sequences were submitted to GenBank (Table A1).

### Phylogenetic analyses

Sequence data for 60 species included in the phylogenetic analyses were retrieved from GenBank, originally published by Liu et al. (2019), Ya et al. (2023), Zhang et al. (2024a, 2024b), Xiong et al. (2025), and Zhu et al. (2025), or were newly generated in this study. Sequence editing was conducted using Sequencher v. 4.1.4 (Gene Codes, Ann Arbor, Michigan, USA), and alignment was performed with MAFFT v. 7.475 (Katoh and Standley 2013) under default parameters. Prior to combining the datasets, congruence between the nrITS and the four plastid markers was checked. The incongruence length difference (ILD) test was used to quantify the conflicts between nuclear DNA and plastid DNA data in PAUP v. 4.0a169 (Darlu and Lecointre 2002; Cummings 2004). Phylogenetic analyses were conducted on the combined matrix, with six species of Aeridinae (*Luisia* Gaudich., *Saccolabium* Blume, *Holcoglossum* Schltr., and *Pomatocalpa* Breda) designated as outgroups following Liu et al. (2019) and Zhang et al. (2024a). The best-fit nucleotide substitution model (GTR+I+G) was selected using jModelTest v. 2.1.6 (Posada 2008) based on the corrected Akaike Information Criterion (AICc). Maximum likelihood (ML) and Bayesian inference (BI) approaches were used for tree reconstruction. BI was run in MrBayes v. 3.2.7a (Ronquist and Huelsenbeck 2003) with two independent Markov chain Monte Carlo (MCMC) runs, each lasting 20 million generations and sampling every 1,000 generations. The first 25% of sampled trees were discarded as burn-in, and the remaining trees were used to build a majority-rule consensus tree. ML analysis was carried out with IQ-TREE v. 1.4.2 (Nguyen et al. 2014), and branch support was assessed using 2,000 bootstrap replicates. The resulting trees were visualized using Chiplot (Xie et al. 2023).

## Results

The aligned nrITS dataset comprised 687 nucleotides, of which 201 were variable. The combined plastid dataset included four markers (*mat*K: 809 bp; *psb*A–*trn*H: 680 bp; *psb*M–*trn*D: 957 bp; *trn*L–F: 1,010 bp), totaling 3,456 bp in length with 213 variable sites. The ILD test (*P* = 0.15) showed that the plastid DNA and nrITS were suitable for combined analysis. In agreement with earlier studies, *Gastrochilus* was recovered as a well-supported monophyletic group, with high posterior probabilities (PP) and bootstrap probabilities (BP). Likewise, the previously recognized subdivision within the genus was corroborated, as the clades corresponding to the six sections received strong support (Fig. 1). The two accessions (*ZJY332* and *ZJY333*) of *G. immaculatus*, collected from two sites, formed a well-supported lineage (BI/ML = 1/100; Fig. 1). This lineage was sister to the clade comprising three accessions of *G. fargesii* (BI/ML = 1/95; Fig. 1). Among the latter, the accession *S18942* from Ya et al. (2023) clustered with the two additional accessions newly obtained in this study, i.e., *ZJY331* (from its type locality, Chengkou County, Chongqing) and *ZJY335* (from Jinfo Mountain, Nanchuan, Chongqing). The subclade comprising *G. immaculatus* and *G. fargesii* was further nested with other species of *G.* sect. *Acinacifolii* (Fig. 1).

Morphologically, *Gastrochilus immaculatus* showed a floral size similar to that of *G. fargesii* and *G. guangtungensis* Z. H. Tsi. However, it was closer to *G. fargesii* than to *G. guangtungensis* in other floral and vegetative characters. Their morphological comparisons are summarized in Table 1.

**Table 1.** Morphological comparisons of *Gastrochilus immaculatus* with two related species of *G.* sect. *Acinacifolii*.

**Character**	***G. immaculatus***	***G. fargesii***	***G. guangtungensis***
Stem length	2.0–8.0 cm	3.0–13.0 cm	6.0–17.0 cm
Leaves	Broad falcate-oblong, 2.5–7.4 × 0.8–1.5 cm	Narrowly falcate-linear, 2.0–4.5 × 0.4–0.6 cm	Falcate-oblong or oblong, 4.5–9.0 × 0.6–1.1 cm
Flowers	ca. 1.2 × 1.4 cm, golden yellow without spots	ca. 0.9 × 1.2 cm, yellowish-green with purplish-red spots	ca. 1.0 × 1.2 cm, yellow with purplish-red spots
Dorsal sepal	Elliptic, 4.6–6.8 × 3.6–4.5 mm, golden yellow without spots	Oblong-elliptic, ca. 4.0 × 2.0 mm, yellowish green with purplish-red spots	Obovate, 5.0–6.0 × 2.2–2.7 mm, yellow with purplish-red spots
Lateral sepals	Obovate, 5.0–6.8 × 3.2–4.5 mm, golden yellow without spots	Similar to dorsal sepal	Similar to dorsal sepal
Petals	Obovate, 4.8–6.5 × 3.2–4.2 mm, golden yellow without spots	Narrowly oblong, ca. 3.0 × 1.5 mm, yellowish green with purplish-red spots	Obovate, 4.5–5.8 × 2.0–2.5 mm, yellow with purplish-red spots
Epichile (lip lamina)	Broadly triangular, 5.8–7.2 × 3.0–4.5 mm, the golden yellow cushion without spots	Transversely oblong, broadly triangular, 2.0–3.0 × 4.0–4.5 mm, the yellowish-white cushion with purplish-red spot	Subovate-triangular, ca. 2.5 × 3.0 mm, the yellow cushion with purplish-red spot
Hypochile	Hypochile subconic, golden yellow, 4.8–6.5 mm tall, 4.2–5.0 × 3.0–3.6 mm in diam., apex acute	Hypochile subconic, yellowish-white, ca. 2.2 mm tall, ca. 3.0 mm in diam., apex obtuse	Hypochile subglobose, white, ca. 3.5 mm tall, ca. 4.5 mm in diam., apex obtuse
Flowering season	September to October	May to June	October
Distribution	Qijiang, Chongqing	Chengkou, Nanchuan, Chongqing; Zunyi, Libo, Guizhou	Wengyuan, Guangdong

## Discussion

The two newly generated accessions of *Gastrochilus immaculatus* were almost identical in the combined ITS and four plastid markers, although they were from different sites separated by cliffs in a deep limestone valley ca. 3.5 km long. This confirms that they both belong to the same species. As previously hypothesized, the general falcate-oblong leaves and nearly triangular and erose-margined epichile of *G. immaculatus* place it in *G.* sect. *Acinacifolii* (Zhang et al. 2024a). The results of phylogenetic analysis based on molecular data support the hypothesis that *G. immaculatus* is a member of this section and sister to *G. fargesii*, to which it is morphologically most similar. Its morphological differences (as shown in Table 1 and the following diagnosis) from *G. fargesii* are relatively stable in stem length, leaf width, and flower color. It is therefore concluded that *G. immaculatus* merits recognition as a new epiphytic orchid species.

With the discovery of *Gastrochilus immaculatus*, the number of species ascribed to *G.* sect. *Acinacifolii* amounts to 13 species (Zhang et al. 2024a). To date, nine of the known species in this section have been sampled for phylogenetic studies, and all were resolved in a strongly supported clade within the genus *Gastrochilus* (Liu et al. 2019; Zhang et al. 2024a, 2024b; Zhu et al. 2025). Increased samples by recent field investigations and comparative morphological studies (Liu et al. 2019; Zhang et al. 2024a) have both revealed the distributions of two species reaching southern China (*G. acutifolius* (Lindl.) Kuntze from eastern India to southwestern Yunnan and *G. minutiflorus* Aver. from Vietnam to southeast Yunnan) and reinstated the identity of *G. somai* (Hayata) Hayata as a separate species rather than a synonym of *G. japonicus* (Makino) Schltr. (Tsi 1999; Chen et al. 2009). *G. fargesii* is the only species in this section distributed in northeast Chongqing, Southwest China; its type materials were collected from the Daba Mountains in Chengkou County. It was formerly reported to have a distributional range extending either from the Daba Mountains to western Sichuan (Qionglai Mountains) (Tsi 1999) or as far as southeastern Yunnan (Chen et al. 2009). A review of those specimens identified as this species during previous studies (see Zhang et al. 2024a, 2024b; Zhu et al. 2025) led to the exclusion of its reported distributions in both Sichuan and Yunnan due to misidentifications, while confirming its presence in southern Chongqing (Nanchuan) and northern (Zunyi) and southeastern Guizhou (Libo) (Fig. 4). However, this still indicates that *G. fargesii* is isolated from other members of this section. The three sites of *G. immaculatus* are not far from a population of *G. fargesii* in Nanchuan (southern Chongqing) (Yi et al. 2010), and the two species show both morphological and phylogenetic affinities. This may suggest that members of the section continued to diversify as they expanded northwards along the mountain ranges bordering the Sichuan Basin, although most species in this section were found farther south (Tsi 1996, 1999; Chen et al. 2009; Liu et al. 2019; Zhang et al. 2024a). Further explorations in adjacent regions may identify additional species in this section or reveal overlooked species diversity among those tree-trunk epiphytic orchids.

### Taxonomic treatment

#### Gastrochilus immaculatus

Taxon classificationPlantaeAsparagalesOrchidaceae

Jun Y.Zhang & F.Chen sp. nov.

33502E14-D306-5CB0-BFC0-71CB8E119D65

urn:lsid:ipni.org:names:77413061-1

Figs 2A, 2B, 3

##### Type.

China • Chongqing: Heishan Valley, Qijiang, growing on tree trunks in evergreen broad-leaved forests; elev. ca. 870 m, 28°52'41"N, 107°01'39"E, in flower, 26 September 2025, *Jun-Yi Zhang & Feng Chen ZJY211* (holotype CDBI!; isotype CQNM!)

##### Diagnosis.

It morphologically resembles *G. fargesii* but differs from the latter by its shorter stem (2.0–8.0 vs. 3.0–13.0 cm), broader falcate-oblong leaves (vs. narrowly falcate-linear), and almost pure golden-yellow flowers (vs. yellowish-green with purplish-red spots). Additionally, its hypochile has a rather acute apex (vs. obtuse apex).

**Figure 2.**
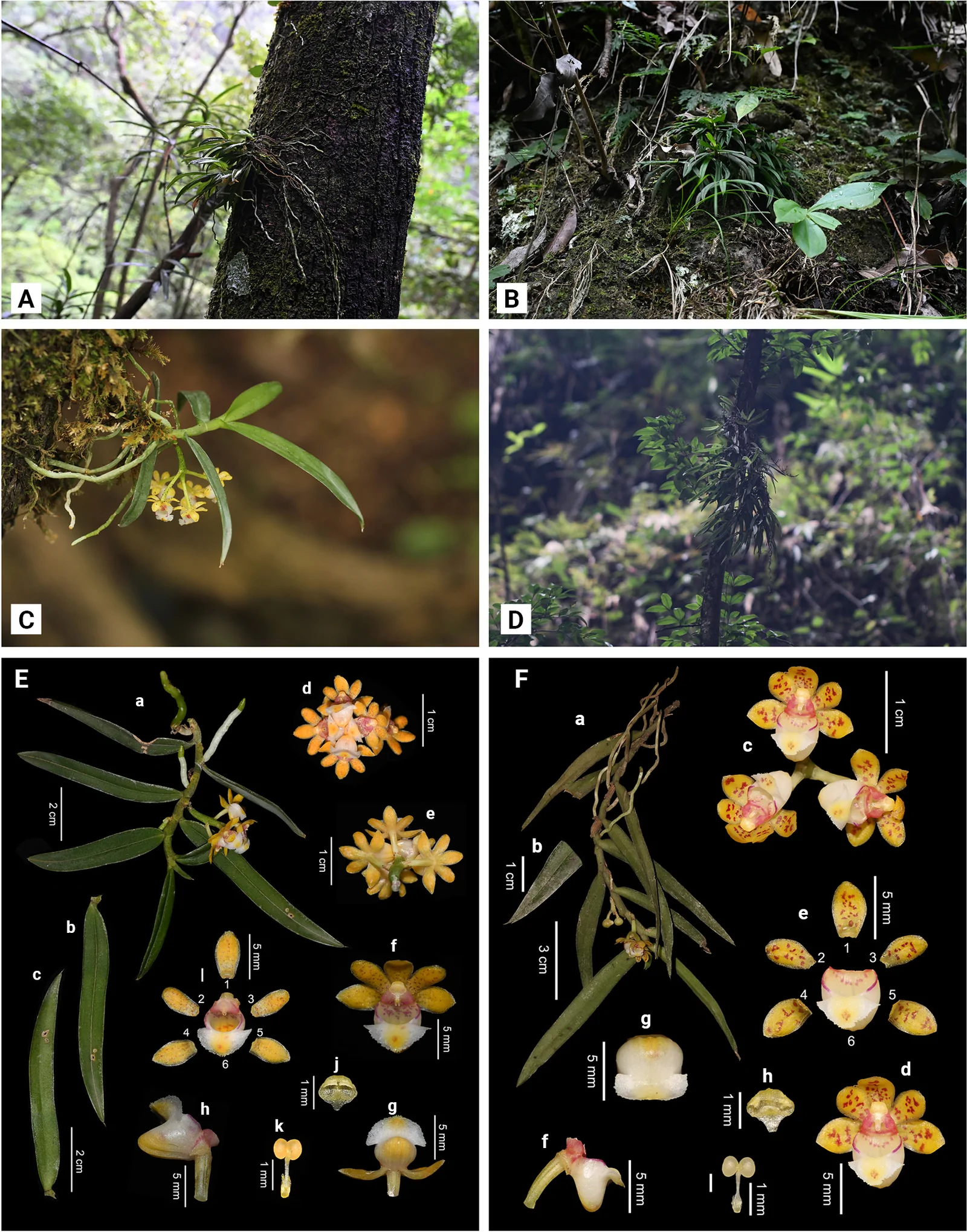
Comparison of two sister species of *Gastrochilus*. **A**. Flowering plants of *G. immaculatus* growing on tree trunks from the Jiaozigou subpopulation. **B**. Flowering plants of *G. immaculatus* growing on rock walls from the Houwangdong subpopulation. **C**. Flowering plants of *G. fargesii* growing on tree trunks from Jinfo Mountain, Nanchuan (southern Chongqing). **D**. Flowering plants of *G. fargesii* growing on tree branches from Chengkou (northeastern Chongqing). **E**. Morphological details of *G. fargesii* from Jinfo Mountain, Nanchuan (southern Chongqing) [**a**. flowering plant; **b**. leaves (front view); **c**. leaves (abaxial view); **d**. inflorescences (front view); **e**. inflorescences (abaxial view); **f**. flowers (front view); **g**. flowers (side view); **h**. hypochile (side view); **i**. dissection of a flower (I1: dorsal sepal; I2–3: lateral sepals; I4–5: petals; I6: labellum); **j**. anther cap; **k**. pollinarium with pollinia]. **F**. Morphological details of *G. fargesii* from Chengkou (northeastern Chongqing) [**a**. flowering plant; **b**. leaf apex (front view); **c**. inflorescences; **d**. flowers (front view); **e**. dissection of a flower (E1: dorsal sepal; E2–3: lateral sepals; E4–5: petals; E6: labellum); **f**. hypochile (side view); **g**. hypochile (abaxial view); **h**. anther cap; **i**. pollinarium with pollinia]. Images **A, B**. Photographed by Feng Chen; **C**. photographed by Ce-Hong Li; **D, F**. photographed by Qiang Liu; **E**. photographed by Jun-Yi Zhang.

**Figure 3.**
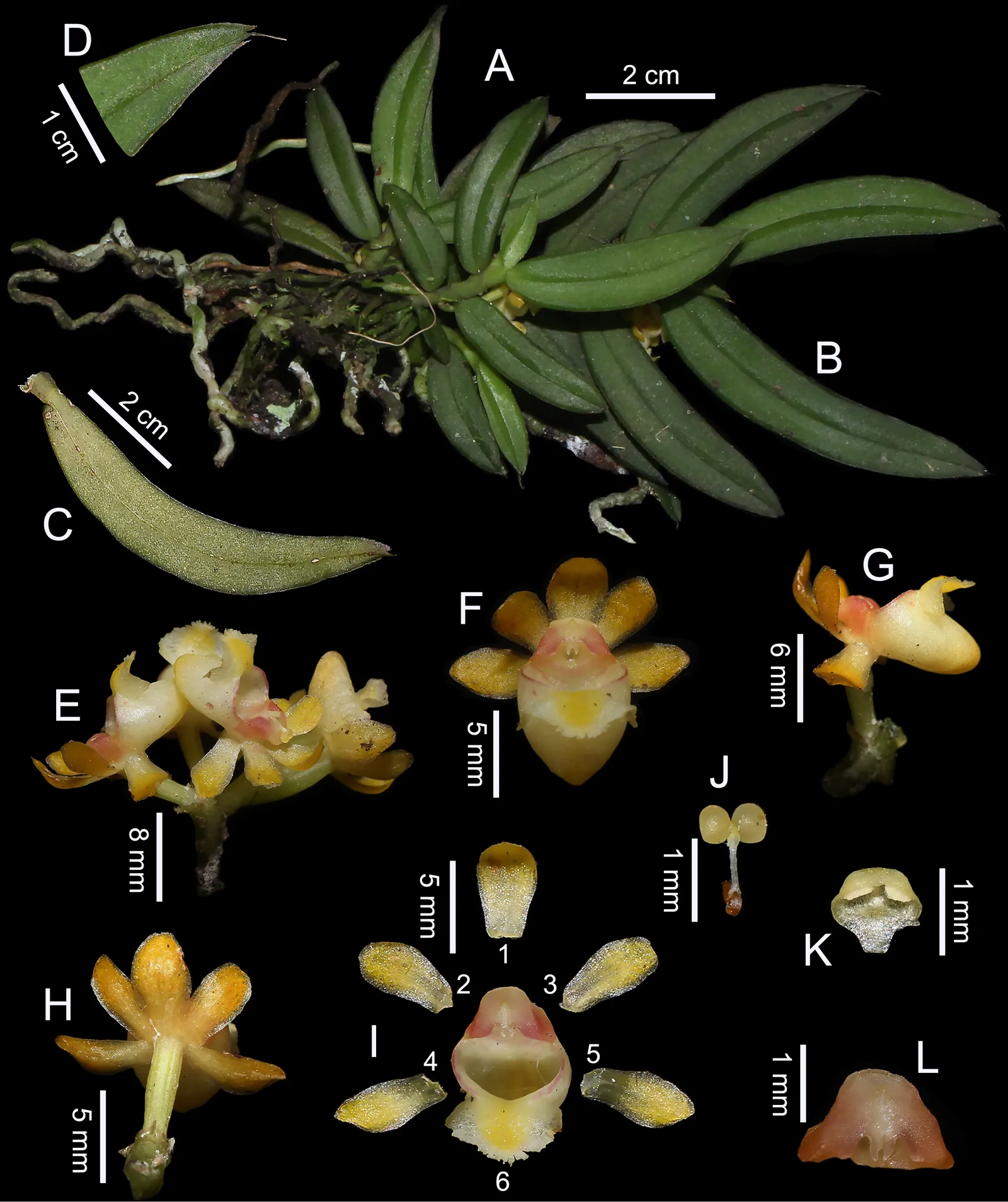
*Gastrochilus immaculatus*. **A**. Flowering plant; **B**. leaves (front view); **C**. leaves (abaxial view); **D**. leaf apex (front view); **E**. inflorescences; **F**. flowers (front view); **G**. flowers (side view); **H**. flowers (abaxial view); **I**. dissection of a flower (I1: dorsal sepal; I2–3: lateral sepals; I4–5: petals; I6: labellum); **J**. pollinarium with pollinia; **K**. anther cap; **L**. stigma. Images **A–L**. Photographed by Feng Chen.

##### Description.

Tree epiphytes, pendent, 4.2–12.5 cm long. Roots vermiform, 6.0–20.0 cm long, ca. 2.0 mm in diameter. Stem sometimes branched, 2.0–8.0 cm long, ca. 3.0 mm in diameter, with many leaves. Leaves distichous, blade thickly fleshy, falcate-oblong, 2.5–7.4 × 0.8–1.5 cm, apex acute and with two or three short awns. Inflorescences several, subumbellate, 2–5-flowered; peduncle curved upward and thickened, 3.0–8.0 mm long, proximally covered with one sheath; floral bracts ovate-lanceolate, 0.6–1.2 mm long, apex acute; pedicellate ovary, 0.5–0.9 cm long. Flowers spreading, ca. 1.2 × 1.4 cm, golden yellow without spots; sepals and petals golden yellow on both sides; dorsal sepals elliptic, 4.6–6.8 × 3.6–4.5 mm, apex obtuse; lateral sepals obovate, 5.0–6.8 × 3.2–4.5 mm, apex obtuse; petals obovate, 4.8–6.5 × 3.2–4.2 mm, apex obtuse, base narrowed; lip with an epichile and a saccate hypochile; epichile broadly triangular, 5.8–7.2 × 3.0–4.5 mm, adaxially glabrous, with a golden-yellow cushion extending to front wall of hypochile, margin ± erose, apex obtuse; hypochile subconic, golden yellow, 4.8–6.5 mm tall, 4.2–5.0 × 3.0–3.6 mm in diam., acute at the apex; column cylindrical, ca. 0.8 mm; anther cap galeate with two chambers, ca. 0.9 × 0.6 mm; rostellum bilobed; pollinarium ca. 0.7 mm long; pollinia 2, ca. 0.3 × 0.2 mm, yellow, subglobular, porate; stigma deeply sunken, inverted V-shaped, ca. 0.3 mm long, pink. Fruits not seen.

##### Habitat and distribution.

**Figure 4.**
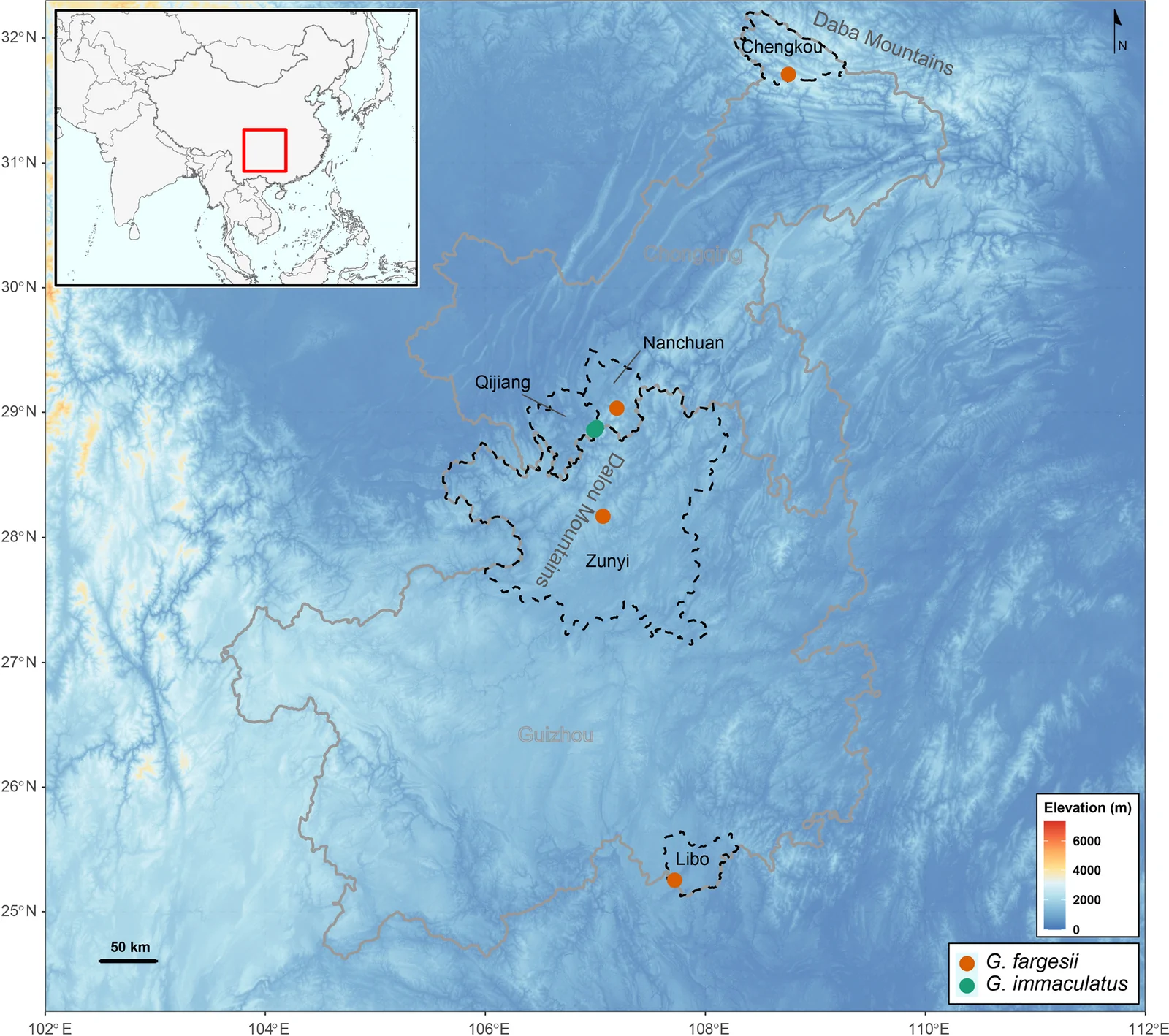
Distribution map of *Gastrochilus immaculatus* and its sister species, *G. fargesii*.

*Gastrochilus immaculatus* is currently known only from Heishan Valley, Qijiang District, Chongqing, southwestern China (Fig. 4). Three subpopulations are currently known, located at Jiaozigou, Heishangu, and Houwangdong in Heishan Town, respectively. It is epiphytic on tree trunks or rock walls in evergreen broad-leaved forests at elevations between 850 and 1,030 m.

##### Conservation status.

The three known subpopulations comprise a total of over 50 mature individuals. Based on the IUCN Red List Categories and Criteria (IUCN 2012), its area of occupancy (AOO) is estimated to be 8 km^2^ (calculated using the standard 2 × 2 km grid cell method), and its extent of occurrence (EOO) is ca. 1.3 km^2^ (reported as 8 km^2^ following IUCN guidelines where EOO<AOO). Because all three subpopulations are restricted to a single valley subject to continuous anthropogenic disturbance and habitat degradation associated with tourism, they represent a single location. Furthermore, a continuous decline in the quality and extent of its habitat has been observed. Although the total number of mature individuals exceeds 50 (which precludes evaluation under Criterion D for CR), the geographical range parameters strictly meet the thresholds for Critically Endangered under Criterion B. Therefore, *Gastrochilus immaculatus* is formally assessed as Critically Endangered (CR): CR B1ab(iii)+2ab(iii). Although further systematic field investigations in adjacent potential habitats in Guizhou Province are recommended, the present assessment strictly reflects its conservation status based on all currently available field data.

##### Phenology.

Flowering from September to October.

##### Etymology.

The specific epithet ‘*immaculatus*’ refers to the spotless sepals, petals, and lip in the flower of this new orchid. Its Chinese name is proposed as *su xin peng ju lan* (素心盆距兰).

##### Other specimens examined.

*Gastrochilus fargesii*. China • Chongqing [previous including in Sichuan]: Chengkou Xian [Tschen Keou], Mingzhong [Moung Moung Ky (蒙蒙溪)], 1600 m, 27 August 1893, *P. G. Farges 1236* (Isotype: IBSC!); Nanchuan County, Houhe, Jinfo Mountain, epiphytic on trunk, 680 m, 29 August 2007, *Yi Si Rong 075631* (IMC!), ibid., 720 m, 03 July 2017, *Qiang Liu 457* (HITBC!).

## Supplementary Material

XML Treatment for Gastrochilus immaculatus

## Appendix 1

**Table A1.** Voucher information and GenBank accession numbers of DNA sequences of *Gastrochilus* used in phylogenetic inference.

**Taxa**	**Voucher**	**Location**	**nrITS**	***mat*K**	***psb*A–*trn*H**	***trn*L–F**	***psb*M–*trn*D**
*G. acaulis* (Lindl.) Kuntze	—	—	KM583455	KM583465	—	—	—
*G. acinacifolius* Z.H.Tsi	*Z. J. Liu 3316*	CHINA	KJ733412	KJ733569	KJ733492	KJ733649	—
*G. acinacifolius* Z.H.Tsi	*Q. Liu 62*	CHINA: Hainan (Baomeiling)	MK357118	MK357138	MK357160	MK357208	MK357216
*G. acinacifolius* Z.H.Tsi	*ZJY289*	CHINA: Yunnan (Malipo)	OQ566796	OQ575562	OQ575590	OQ575530	OQ575619
*G. acutifolius* (Lindl.) Kuntze	*Q. Liu 05*	CHINA: Yunnan (Jingmai)	—	MK357140	MK357162	—	MK357230
*G. acutifolius* (Lindl.) Kuntze	*JK-DEBCR-mat-31*(JK)	—	MW475270	MW433889	—	—	—
*G. affinis* (King & Pantl.) Schltr.	*Q. Liu 97*	CHINA: Yunnan (Fugong)	—	MK357141	MK357163	—	MK357141
*G. alatus* X.H.Jin & S.C.Chen	*Q. Liu 98*	CHINA: Yunnan (Bingzhongluo)	—	—	—	—	MK357228
*G. alatus* X.H.Jin & S.C.Chen	*ZJY297*	CHINA: Yunnan	—	OQ575563	OQ575591	OQ575531	OQ575620
*G. armeniacus* Jun Y.Zhang, B.Xu & Yue H.Cheng	*ZJY244*	CHINA: Sichuan (Wenchuan)	OP348889	OP373113	OP373119	OP373128	OP373123
*G. armeniacus* Jun Y.Zhang, B.Xu & Yue H.Cheng	*ZJY285*	CHINA: Sichuan (Wenchuan)	OP348888	OP373114	OP373118	OP373127	OP373124
*G. balangshanensis* Jun Y.Zhang, B.Xu & Yue H.Cheng	*ZJY317*	CHINA: Sichuan (Wenchuan)	PP960151	PP944493	PP944495	PP944499	PP944497
*G. balangshanensis* Jun Y.Zhang, B.Xu & Yue H.Cheng	*ZJY318*	CHINA: Sichuan (Wenchuan)	PP960152	PP944494	PP944496	PP944500	PP944498
*G. bellinus* (Rchb.f.) Kuntze	*Q. Liu 52*	CHINA: Yunnan (Lancang)	KY966597	KY966884	—	—	—
*G. bellinus* (Rchb.f.) Kuntze	*Q. Liu 53*	CHINA: Yunnan (Mengsong)	MK357123	MK357142	MK357164	MK357202	MK357241
*G. bellinus* (Rchb.f.) Kuntze	*ZJY279*	CHINA: Yunnan (Mengla)	OQ566799	OQ575564	OQ575592	OQ575534	OQ575621
*G. bernhardtianus* J.D.Ya & D.Z.Li	*20CS19022*	CHINA: Yunnan (Lijiang)	OR073404	OR772951	OR772951	OR772951	OR772951
*G. bernhardtianus* J.D.Ya & D.Z.Li	*20CS19023*	CHINA: Yunnan (Lijiang)	OR073405	OR002167	OR002167	OR002167	OR002167
*G. bigibbus* (Rchb.f. ex Hook.f.) Kuntze	*Li 009*	—	—	MN124439	MN124439	MN124439	MN124439
*G. calceolaris* D.Don	*Z. J. Liu 3769*	CHINA	KF545874	KF545885	KF545865	—	KF545896
*G. calceolaris* D.Don	*Q. Liu 94*	CHINA: Yunnan (Jingdong)	MK357126	MK357144	MK357169	MK357205	MK357233
*G. calceolaris* D.Don	*ZJY281*	CHINA: Yunnan (Malipo)	—	OQ575565	OQ575593	OQ575535	OQ575622
*G. changjiangensis* Q.Liu & M.Z.Huang	*Q. Liu 45*	CHINA: Hainan (Changjiang)	MK357124	—	MK357166	—	MK357236
*G. ciliaris* F.Maekawa	*Q. Liu 88*	CHINA: Fuzhou	—	MK357148	MK357173	—	MK357225
*G. ciliaris* F.Maekawa	*ZJY299*	CHINA: Taiwan	—	OQ575566	OQ575594	—	OQ575623
*G. dasypogon* (Lindl.) Kuntze	*Q. Liu 30*	CHINA: Hainan (Baomeiling)	MK357129	MK357149	MK357181	MK357197	MK357219
*G. deminutus* J.M.H.Shaw	—	—	KY966600	KY966887	—	—	—
*G. distichus* (Lindl.) Kuntze	*Z. J. Liu 4755*	CHINA	KJ733414	KJ733571	KJ733494	KJ733651	—
*G. distichus* (Lindl.) Kuntze	*ZJY309*	CHINA: Yunnan (Malipo)	OQ566800	OQ575567	OQ575595	OQ575536	—
*G. fargesii* (Kraenzl.) Schltr.	*S18942*	CHINA: Chongqing	—	OR002175	OR002175	OR002175	OR002175
*G. fargesii* (Kraenzl.) Schltr.	*ZJY331*	CHINA: Chongqing (Chengkou)	PZ281538	PZ282778	PZ282782	PZ282786	PZ282790
*G. fargesii* (Kraenzl.) Schltr.	*ZJY335*	CHINA: Chongqing (Nanchuan)	PZ281539	PZ282779	PZ282783	PZ282787	PZ282791
*G. formosanus* (Hayata) Hayata	*Z. J. Liu 4256*	CHINA	KJ733416	KJ733573	KJ733495	KJ733653	—
*G. formosanus* (Hayata) Hayata	*Q. Liu 91*	CHINA: Yunnan (Gongshan)	—	—	MK357174	—	MK357226
*G. formosanus* (Hayata) Hayata	*ZJY276*	CHINA: Hubei (Enshi)	OQ566801	OQ575568	OQ575596	OQ575537	OQ575624
*G. fuscopunctatus* (Hayata) Hayata	*Q. Liu 86*	CHINA: Taiwan (Hualian)	—	MK357150	MK357171	MK357192	MK357231
*G. fuscopunctatus* (Hayata) Hayata***	—	—	—	KX871233	KX871233	KX871233	KX871233
*G. gongshanensis* Z.H.Tsi	*ZJY295*	CHINA: Yunnan (Malipo)	—	OQ575569	OQ575597	OQ575538	OQ575625
*G. guangtungensis* Z.H.Tsi	*Z. J. Liu 4127*	CHINA	KJ733417	KJ733574	KJ733496	KJ733654	—
*G. guangtungensis* Z.H.Tsi	*ZJY293*	CHINA: Yunnan	OQ566802	OQ575570	OQ575598	OQ575539	OQ575626
*G. heminii* M.Liao, B.Xu & Yue.H.Cheng	*ZJY241*	CHINA: Sichuan (Wenchuan)	ON286752	ON331126	ON331128	ON331130	ON331132
*G. heminii* M.Liao, B.Xu & Yue.H.Cheng	*ZJY283*	CHINA: Sichuan (Wenchuan)	ON286753	ON331127	ON331129	ON331131	ON331133
***G. immaculatus* Jun Y.Zhang & F.Chen**	***ZJY332***	**CHINA: Chongqing (Qijiang)**	**PZ281540**	**PZ282780**	**PZ282784**	**PZ282788**	**PZ282792**
***G. immaculatus* Jun Y.Zhang & F.Chen**	***ZJY333***	**CHINA: Chongqing (Qijiang)**	**PZ281541**	**PZ282781**	**PZ282785**	**PZ282789**	**PZ282793**
*G. intermedius* (Griff. ex Lindl.) Kuntze	*Q. Liu 38*	CHINA: Yunnan (Yinchang)	MK357121	MK357151	MK357172	MK357190	MK357213
*G. japonicus* (Makino) Schltr.	*Q. Liu 87*	CHINA: Taiwan (Pingdong)	KF545875	KF545886	KF545866	KF545897	—
*G. japonicus* (Makino) Schltr.	—	—	—	KX871236	KX871236	KX871236	KX871236
*G. jiuzhaigouensis* Jun Y.Zhang, D.L.Zhu & Y.Zhang	*ZJY329*	CHINA: Sichuan (Jiuzhaigou)	PV643100	PV645752	PV645754	PV645758	PV645756
*G. jiuzhaigouensis* Jun Y.Zhang, D.L.Zhu & Y.Zhang	*ZJY330*	CHINA: Sichuan (Jiuzhaigou)	PV643101	PV645753	PV645755	PV645759	PV645757
*G. kadooriei* Kumar et al.	*ZJY268*	CHINA: Yunnan (Malipo)	OQ566803	OQ575571	OQ575599	OQ575540	OQ575627
*G. lihengiae* J.D.Ya, H.Jiang & D.Z.Li	*22CS21828*	CHINA: Yunnan (Gongshan)	OR073408	OR002168	OR002168	OR002168	OR002168
*G. lihengiae* J.D.Ya, H.Jiang & D.Z.Li	*23CS24145*	CHINA: Yunnan (Gongshan)	OR073408	OR002169	OR002169	OR002169	OR002169
*G. linearifolius* Z.H.Tsi & Garay	*Q. Liu 711*	MYANMA: Hkakaborazi	MK357133	MK357136	MK357187	MK357194	MK357229
*G. linii* Ormerod	*Q. Liu 92*	CHINA: Fuzhou	—	MK357152	MK357176	MK357198	MK357224
*G. malipoensis* X.H.Jin & S.C.Chen	*Q. Liu 71*	CHINA: Yunnan (Nanwenghe)	—	MK357147	MK357177	MK357200	MK357235
*G. malipoensis* X.H.Jin & S.C.Chen	*ZJY288*	CHINA: Yunnan (Malipo)	OQ566804	OQ575572	OQ575600	OQ575541	OQ575628
*G. matsuran* (Makino) Schltr.	—	—	KT338700	—	—	—	—
*G. minjiangensis* Jun Y.Zhang, B.Xu & Yue H.Cheng	*ZJY243*	CHINA: Sichuan (Wenchuan)	OP348887	OP373112	OP373117	OP373126	OP373122
*G. minjiangensis* Jun Y.Zhang, B.Xu & Yue H.Cheng	*ZJY284*	CHINA: Sichuan (Wenchuan)	OQ566806	—	—	OQ575542	—
*G. minutiflorus* Aver.	*Q. Liu 37*	CHINA: Yunnan (Malipo)	—	MK357153	MK357179	—	MK357215
*G. minutiflorus* Aver.	*ZJY287*	CHINA: Yunnan (Malipo)	OQ566807	OQ575573	OQ575601	OQ575544	OQ575629
*G. nanchuanensis* Z.H.Tsi	*ZJY296*	CHINA: Chongqing (Nanchuan)	OQ566808	OQ575574	OQ575602	OQ575545	OQ575630
G. obliquus var. obliquus (Lindl.) Kuntze	*Q. Liu 708*	MYANMA: Hponkanrazi Wildlife Sanctuary	MK357131	MK357137	KJ733498	KJ733656	MK357211
G. obliquus var. obliquus (Lindl.) Kuntze	*Q. Liu 44*	CHINA: Yunnan (Yinchang)	MK357130	MK357154	MK357182	MK357195	MK357218
*G. obovatifolius* C.Xiong, X.Y.Fu & S.R.Yi	*XC24008*	CHINA: Chongqing (Chengkou)	PP949380	PP942372	PP942372	PP942372	PP942372
*G. suavis* Seidenf.	*ZJY277*	CHINA: Yunnan	OQ566809	OQ575575	OQ575603	OQ575546	OQ575631
*G. suavis* Seidenf.	*ZJY282*	CHINA: Yunnan (Maguan)	OQ566810	OQ575576	OQ575604	OQ575547	OQ575632
*G. platycalcaratus* (Rolfe) Schltr.	*Q. Liu 19*	CHINA: Yunnan (Yinchang)	MK357122	—	MK357175	—	MK357222
*G. platycalcaratus* (Rolfe) Schltr.	*ZJY300*	CHINA: Yunnan (Mengla)	OQ566811	—	OQ575605	OQ575548	OQ575633
*G. prionophyllus* H.Jiang, D.P.Ye & Q.Liu	*ZJY291*	CHINA: Yunnan	OQ566812	OQ575577	OQ575606	OQ575549	OQ575634
*G. pseudodistichus* (King & Pantl.) Schltr.	*ZJY308*	CHINA: Yunnan (Malipo)	OQ566813	OQ575578	OQ575607	OQ575550	OQ575635
*G. pseudodistichus* (King & Pantl.) Schltr.	*ZJY290*	CHINA: Yunnan (Maguan)	OQ566814	OQ575579	OQ575608	OQ575551	OQ575636
*G. rantabunensis* S.Chow ex T.P.Lin	*Q. Liu 89*	CHINA: Taiwan (Taipei)	—	MK357155	MK357184	MK357193	MK357223
*G. rantabunensis* S.Chow ex T.P.Lin	*ZJY303*	CHINA: Taiwan	OQ566815	OQ575580	OQ575609	OQ575552	OQ575637
*G. raraensis* Fukuy.	*Z. J. Liu 4798*	CHINA	KJ733420	KJ733577	KJ733499	KJ733657	—
*G. setosus* Aver. & Vuong	*ZJY304*	Vietnam	OQ566816	OQ575581	OQ575610	OQ575553	—
*G. sinensis* Z.H.Tsi	*ZJY228*	CHINA: Zhejiang (Hangzhou)	OM985813	OK042953	OK172399	OK172401	OQ575646
*G. sinensis* Z.H.Tsi	*S1109*	CHINA: Sichuan (Wenchuan)	OP348890	OP373115	OP373120	OP373129	OP373125
*G. somai* (Hayata) Hayata	*Q. Liu 36*	CHINA: Fujian (Pingnan)	MK357128	—	MK357180	—	MK357220
*G. somai* (Hayata) Hayata	*Li 073*	—	—	MN124436	MN124436	MN124436	MN124436
*G. sororius* Schltr.	—	—	ERR7622420	ERR7622420	ERR7622420	ERR7622420	
*G. wenshanensis* Q.Liu, D.P.Ye & X.H.Jin	*ZJY292*	CHINA: Yunnan (Maguan)	OQ566820	OQ575586	OQ575615	OQ575558	OQ575642
*G. sumartranus* J.J.Sm.	*ZJY280*	Vietnam	OQ566818	OQ575583	OQ575612	OQ575555	OQ575639
*G. sumartranus* J.J.Sm.	*ZJY294*	Vietnam	OQ566819	OQ575584	OQ575613	OQ575556	OQ575640
*G. tianbaoensis* Q.Liu & Y.H.Tan	*ZJY275*	CHINA: Yunnan (Malipo)	—	OQ575585	OQ575614	OQ575557	OQ575641
*G. tianbaoensis* Q.Liu & Y.H.Tan	*Q. Liu 63*	CHINA: Yunnan (Malipo)	MK357120	MK357157	MK357186	MK357207	MK357214
*G. wolongensis* Jun Y.Zhang, B.Xu & Yue H.Cheng	*ZJY240*	CHINA: Sichuan (Wenchuan)	OM985810	OK172400	OK172402	OK172404	OK172403
*G. wolongensis* Jun Y.Zhang, B.Xu & Yue H.Cheng	*S1108*	CHINA: Sichuan (Wenchuan)	OM985811	OM974209	OM974211	OM974210	OQ575647
*G. xuanenensis* Z.H.Tsi	*ZJY298*	CHINA: Guizhou	—	OQ575587	OQ575616	OQ575559	OQ575643
*G. yunlongensis* W.H.Rao, L.J.Chen & Z.J.Liu	*ZJY301*	CHINA: Yunnan	—	OQ575588	OQ575617	OQ575560	OQ575644
*G. yunnanensis* Schltr.	*Q. Liu 60*	CHINA: Yunnan (Mengsong)	MK165469	MK357158	MK357185	—	MK357212
*G. yunnanensis* Schltr.	*ZJY278*	CHINA: Yunnan (Xinping)	OQ566821	OQ575589	OQ575618	OQ575561	OQ575645
*G. zhenyuanensis* Q.Liu & D.P.Ye	*Q. Liu 61*	CHINA: Yunnan (Zhenyuan)	MK357127	MK357146	MK357168	MK357199	MK357237
*Holcoglossum amesianum* (Rchb.f.) Christenson	*X.H. Jin 004/006*	CHINA	HQ404389	JF763779	HQ404439	—	—
*H. kimballianum* (Rchb.f.) Garay	*X.H. Jin 017*	CHINA	HQ452901	JF763787	HQ404452	—	—
*Luisia magniflora* Z.H.Tsi & S.C.Chen	*Z. J. Liu 3444*	CHINA	KJ733426	KJ733583	KJ733505	—	—
*Pomatocalpa diffusum* Breda	*TBG145837*	INDONESIA	AB217576	AB217752	—	EF670432	—
*P. spicatum* Breda	*Z. J. Liu 4589*	CHINA	KJ733438	KJ733595	KJ733518	KJ733675	—
*Saccolabium pusillum* Blume	*TBG144220*	INDIA	AB217580	AB217756	—	—	—

## Additional information

### Artificial Intelligence (AI) use

The authors accept full responsibility for the content of the manuscript, including the disclosure of any use of AI.

No AI tools were used in the preparation of this manuscript.

### Author ORCIDs

J.-Y. Zhang https://orcid.org/0000-0001-8325-5954

M. Liao https://orcid.org/0000-0001-6777-8579

P.-Y. Yang https://orcid.org/0000-0003-0346-0443

Y.-H. Cheng https://orcid.org/0000-0001-6263-0989

D.-X. Yu https://orcid.org/0000-0002-7467-1769

F. Chen https://orcid.org/0000-0003-3398-8900

B. Xu https://orcid.org/0000-0003-3507-9321

### Data availability

All of the data that support the findings of this study are available in the main text.

## References

[B1] Beentje H (2016) The Kew plant glossary: an illustrated dictionary of plant terms, 2^nd^ edn. Kew Publishing, Kew, 184 pp.

[B2] Chen XQ, Tsi ZH, Wood JJ (2009) *Gastrochilus* D. Don. In: Wu ZY, Raven PH, Hong DY (Eds) Flora of China, vol. 25. Science Press, Beijing & Missouri Botanical Garden Press, St. Louis, 491–498.

[B3] Cummings MP (2004) PAUP* [Phylogenetic Analysis Using Parsimony (and Other Methods)]. In: Hancock JM, Zvelebil MJ (Eds) Dictionary of Bioinformatics and Computational Biology. 10.1002/0471650129.dob0522

[B4] Darlu F, Lecointre G (2002) When does the incongruence length difference test fail? Molecular Biology and Evolution 19: 432–437. 10.1093/oxfordjournals.molbev.a004098 11919284

[B5] Don D (1825) Prodromus Florae Nepalensis. J. Gale, London, 256 pp.

[B6] Govaerts R, Campacci MA, Baptista DH, Baptista PJ, George A, Kreutz K, Wood JJ (2021) World Checklist of Orchidaceae. The Board of Trustees of the Royal Botanic Gardens, Kew. http://www.kew.org/wcsp/monocots/ [Accessed: 13 August 2021]

[B7] IUCN (2012) Guidelines for application of IUCN Red List criteria at regional and national levels, version 4.0. IUCN Red List Unit, Gland and Cambridge, 41 pp.

[B8] Katoh K, Standley DM (2013) MAFFT multiple sequence alignment software version 7: Improvements in performance and usability. Molecular Biology and Evolution 30: 772–780. 10.1093/molbev/mst010 PMC3603318 23329690

[B9] Lee CT, Wu JH, Wang YQ, Hsieh SI (2023) *Gastrochilus yehii* sp. nov. (Orchidaceae) from Taiwan. Phytotaxa 587: 053–058. 10.11646/phytotaxa.587.1.7

[B10] Liao M, Cheng YH, Zhang JY, Feng Y, Liu GY, Ye P, Jin SL, Lin HQ, Xu B (2022) *Gastrochilus heminii* (Orchidaceae, Epidendroideae), a new species from Sichuan, China, based on molecular and morphological data. PhytoKeys 215: 95–106. 10.3897/phytokeys.215.91061 PMC9836483 36761093

[B11] Liu Q, Song Y, Jin XH, Gao JY (2019) Phylogenetic relationships of *Gastrochilus* (Orchidaceae) based on nuclear and plastid DNA data. Botanical Journal of the Linnean Society 189: 228–243. 10.1093/botlinnean/boy084

[B12] Liu Q, Wu XF, Zhou SS, Li JW, Jin XH (2023) New species and record of *Gastrochilus* (Orchidaceae, Aeridinae) from China and Laos. Phytotaxa 585: 210–224. 10.11646/phytotaxa.585.3.3

[B13] Nguyen L, Schmidt HA, Von-Haeseler A, Minh BQ (2014) IQ-TREE: A fast and effective stochastic algorithm for estimating Maximum-Likelihood phylogenies. Molecular Biology and Evolution 32: 268–274. 10.1093/molbev/msu300 PMC4271533 25371430

[B14] Nguyen VC, Averyanov LV, Maisak TV, Nguyen TLT, Nguyen VK, Truong BV (2022) *Gastrochilus pankajkumarii*, (Aeridinae, Epidendroideae, Orchidaceae) a new lithophytic orchid from southern Vietnam. Taiwania 67: 35–39. 10.6165/tai.2022.67.35

[B15] Posada D (2008) jModelTest: Phylogenetic model averaging. Molecular Biology and Evolution 25: 1253–1256. 10.1093/molbev/msn083 18397919

[B16] POWO (2026) Plants of the World Online. Facilitated by the Royal Botanic Gardens, Kew. Published on the Internet. https://powo.science.kew.org/ [Retrieved: 03 September 2026]

[B17] Pridgeon AM, Cribb PJ, Chase MW, Rasmussen FN (2014) Genera orchidacearum: Epidendroideae, volume 6, Part 3. Oxford University Press, Oxford, 544 pp.

[B18] Rao WH, Liu ZJ, Zhang GQ, Chen XH, Huang J, Chen GZ, Chen LJ (2019) A new epiphytic species of *Gastrochilus* (Orchidaceae: Epidendroideae) from Yunnan, China. Phytotaxa 413: 296–300. 10.11646/phytotaxa.413.4.5

[B19] Ronquist F, Huelsenbeck JP (2003) MrBayes 3: Bayesian phylogenetic inference under mixed models. Bioinformatics (Oxford, England) 19: 1572–1574. 10.1093/bioinformatics/btg180 12912839

[B20] Thiers B (2026) Index Herbariorum: a global directory of public herbaria and associated staff. New York Botanical Garden’s Virtual Herbarium. http://sweetgum.nybg.org/science/ih

[B21] Tsi ZH (1996) A preliminary revision of *Gastrochilus* (Orchidaceae). Guihaia 16: 123–154.

[B22] Tsi ZH (1999) *Gastrochilus* D. Don. In: Tsi ZH (Ed.) Flora Reipublicae Popularis Sinicae, vol. 19. Science Press, Beijing, 399–420.

[B23] Wu XF, Ye DP, Pan B, Lin XQ, Jiang H, Liu Q (2019) Validation of *Gastrochilus prionophyllus* (Vandeae, Orchidaceae), a new species from Yunnan Province, China. PhytoKeys 130: 161–169. 10.3897/phytokeys.130.34555 PMC6728340 31534404

[B24] Xie JM, Chen YR, Cai GJ, Cai RL, Hu Z, Wang H (2023) Tree visualization by one Table (tvBOT): A web application for visualizing, modifying and annotating phylogenetic trees. Nucleic Acids Research 51: W587–W592. 10.1093/nar/gkad359 PMC10320113 37144476

[B25] Xiong C, Fu XY, Tan K, Huang Y, Zhang HJ, Wang YC, Yang YB, Yi SR (2025) *Gastrochilus obovatifolius* (Orchidaceae, Aeridinae), a new species from the Daba Mountains of Chongqing, China. PhytoKeys 252: 25–40. 10.3897/phytokeys.252.133501 PMC11829196 39957784

[B26] Ya JD, Wang WT, Liu YL, Jiang H, Han ZD, Zhang T, Huang H, Cai J, Li DZ (2023) Five new and noteworthy species of Epidendroideae (Orchidaceae) from southwestern China based on morphological and phylogenetic evidence. PhytoKeys 235: 211–236. 10.3897/phytokeys.235.111230 PMC10682981 38033625

[B27] Yi SR, Huang Y, Xiao B, Shen ML, Li J (2010) *Gastrochilus brevifimbriatus* (Orchidaceae, Aeridinae), a new species from Chongqing, China. Novon: A Journal for Botanical Nomenclature 20(1): 113–116. 10.3417/2008013

[B28] Zhang JY, Cheng YH, Liao M, Jin SL, Qu CM, Tan YC, Plenković-Moraj A, Xu B (2022) *Gastrochilus wolongensis* (Orchidaceae): A new species from Sichuan, China, based on molecular and morphological data. Ecosystem Health and Sustainability 8(1): 2101546. 10.1080/20964129.2022.2101546

[B29] Zhang JY, Cheng YH, Liao M, Feng Y, Jin SL, He TM, He H, Xu B (2024a) A new infrageneric classification of *Gastrochilus* (Orchidaceae: Epidendroideae) based on molecular and morphological data. Plant Diversity 46: 435–447. 10.1016/j.pld.2023.08.001 PMC11390603 39280969

[B30] Zhang JY, Cheng YH, Liao M, Jin SL, Lin HQ, Yang PY, He H, Xu B (2024b) *Gastrochilus balangshanensis* (Orchidaceae, Aeridinae), a new subalpine epiphytic orchid from the Mountains of Southwest China. PhytoKeys 247: 123–135. 10.3897/phytokeys.247.130755 PMC11489711 39429518

[B31] Zhou CY, Zeng MY, Wu YW, Li MH (2024) *Gastrochilus pseudodresslerii* (Orchidaceae; Vandeae), a new species from China: evidence from morphological and DNA analysis. Phytotaxa 634: 071–078. 10.11646/phytotaxa.634.1.6

[B32] Zhou ZH, Shi RH, Zhang Y, Jin XH (2021) Orchid diversity in China: Recent discoveries. Plant Diversity 43(5): 341–342. 10.1016/j.pld.2021.07.004 PMC8591207 34816059

[B33] Zhu DL, Zhang JY, Zhang Y, Liao M, He H, Chen K, Xu B (2025) *Gastrochilus jiuzhaigouensis* (Orchidaceae, Epidendroideae), a new epiphytic orchid from Jiuzhaigou World Heritage Site, China. PhytoKeys 265: 25–38. 10.3897/phytokeys.265.159138 PMC12541466 41133110

